# Munir: A Multimodal Smart-Glasses System for Enhancing Human–Computer Interaction for Visually Impaired Individuals

**DOI:** 10.3390/s26123950

**Published:** 2026-06-22

**Authors:** Nora Alhammad, Aljawharah Alsubaie, Rama Alomair, Fajer Alamro, Mashael Alammar

**Affiliations:** College of Computer and Information Sciences, King Saud University, Riyadh 11451, Saudi Arabia; 444201140@student.ksu.edu.sa (A.A.); 444200662@student.ksu.edu.sa (R.A.); 444200800@student.ksu.edu.sa (F.A.); 444200786@student.ksu.edu.sa (M.A.)

**Keywords:** assistive technology, human–computer interaction (HCI), multimodal interaction, smart glasses, visual impairment, face recognition, computer vision, wearable sensors, bilingual interface

## Abstract

Visual impairment affects approximately 2.2 billion people worldwide, yet existing assistive technologies remain fragmented and prohibitively expensive. This paper presents Munir, an integrated multimodal assistive system designed to enhance human–computer interaction through a combination of a mobile application and Bluetooth-enabled smart glasses. Munir leverages a hybrid machine learning architecture to provide inclusive, real-time support for daily living activities. The system integrates ten core capabilities—including face recognition, optical character recognition, and scene description—all accessible through a unified bilingual (Arabic/English) voice interface. By employing on-device processing for biometric tasks, Munir ensures user privacy and trust while maintaining high responsiveness. End-to-end system evaluation on the SCface dataset achieves a 96.69% recognition rate with 0% False Accept Rate. At an estimated first-year total cost of $806, Munir demonstrates a 4–5× cost advantage over commercial alternatives, providing a scalable and affordable multimodal solution for global digital inclusion.

## 1. Introduction

Visual impairment represents one of the most prevalent disabilities globally, affecting approximately 2.2 billion people according to the World Health Organization [1]. These individuals face significant daily challenges including recognizing faces for social interaction, reading text, navigating environments, and maintaining independence in routine activities. Research demonstrates strong correlations between visual impairment and elevated rates of depression, anxiety, and social isolation [2]. Furthermore, educational and employment barriers are exacerbated when assistive technologies prove unavailable or unaffordable.

### 1.1. Limitations of Current Solutions

Existing assistive technologies exhibit critical limitations that restrict their practical utility. Commercial smart glasses such as OrCam MyEye ($4250) and Envision Glasses ($3500) offer text reading and face recognition capabilities [3,4] but are constrained by (1) prohibitive costs that exclude the majority of potential users globally; in contrast to the substantially lower cost structure detailed in Table 1 for the proposed system; (2) absence of integrated emergency alert and safety functionality; (3) limited bilingual support across existing solutions, as reflected in Table 2. Smartphone applications like Microsoft Seeing AI provide free alternatives with competent computer vision [5]; however, continuous smartphone handling fundamentally limits mobility utility, as users must simultaneously control canes or guide dogs. To date, no existing solution combines comprehensive functionality, hands-free operation, Arabic language support, and economic accessibility at a global scale.

### 1.2. Research Contributions

This paper presents Munir, an integrated AI-powered assistive system addressing these gaps through four key contributions:

(1) System Architecture Contribution—Comprehensive Multimodal Integration: While existing solutions such as OrCam MyEye and Envision Glasses address isolated assistive challenges, Munir unifies ten capabilities—face recognition, OCR, color detection, object recognition, scene description, GPS emergency alerts, task reminders, and a bilingual Arabic/English voice interface—within a single hands-free, voice-controlled system. Unlike Seeing AI, which does not support Arabic, and unlike OrCam and Envision, which lack emergency alert functionality, this architectural integration consolidates capabilities absent from existing commercial solutions into a unified platform.

(2) Empirical Contribution—Systematic Face Recognition Model Selection: Rather than adopting a pre-existing model without justification, a systematic closed-set evaluation of three architectures (InsightFace buffalo_l, FaceNet512, and FaceNet) was conducted on a curated VGGFace2 subset under a Single-Gallery-Image constraint—reflecting worst-case enrollment conditions in assistive deployments. InsightFace buffalo_l achieved the highest Top-1 accuracy (91.52%), outperforming FaceNet512 by 25.9 percentage points, providing an empirical basis for model selection specific to this application context.

(3) Design Contribution—Privacy-Preserving On-Device Biometric Processing and Secure Communication: A core architectural decision in Munir is that face recognition inference executes entirely on-device, ensuring that biometric facial data is never transmitted to external servers. This contrasts with solutions such as Seeing AI, which processes facial recognition through cloud-based APIs. To safeguard data-in-transit between the wearable glasses and the mobile application, the system operates over the standard BLE LE Secure Connections mode (introduced in BLE v4.2), which employs Elliptic Curve Diffie–Hellman (ECDH P-256) for key exchange and hardware-accelerated AES-CCM 128-bit encryption at the Link Layer via the ESP32-S3, ensuring all local data streams—including raw microphone audio and JPEG camera frames—are encrypted at the protocol level rather than through a custom application layer, preventing eavesdropping and Man-in-the-Middle (MitM) attacks. Non-biometric features such as object detection and scene description utilize cloud APIs, with users informed of this distinction. This rigorous separation between on-device biometric processing, secure encrypted local transmission, and cloud-based non-biometric features directly addresses privacy concerns for a vulnerable user population.

(4) Engineering Contribution—Flexible Enrollment and Cost-Effective Hardware: Munir introduces a dual-pathway face enrollment mechanism—live capture via the smartphone camera or direct upload from the device gallery—addressing a practical limitation of commercial devices such as OrCam MyEye, which requires the person to be physically present during enrollment. Combined with integration of the open-source Omi Glass Dev Kit, the estimated first-year total cost of $806 represents a 4–5× reduction compared to OrCam MyEye ($4250) and Envision Glasses ($3500).

## 2. Related Work

### 2.1. Assistive Technology for Visual Impairment

Assistive technologies have evolved from mechanical aids to contemporary AI-powered solutions [6]. While traditional aids remain essential, modern solutions increasingly leverage mobile computing and artificial intelligence [7]. Be My Eyes pioneered crowdsourced assistance connecting blind users with sighted volunteers [8], yet this approach depends on volunteer availability and raises privacy concerns. These limitations underscore the need for autonomous AI-based solutions [9].

Commercial smart glasses represent the current state of the art. Google Glass (2013) pioneered consumer smart glasses but failed commercially due to privacy concerns and limited applications [10]. Envision Glasses ($3500) targets assistive applications with text reading and face recognition. Vuzix Blade ($1000) focuses on enterprise augmented reality rather than accessibility. More recent work has demonstrated smart-glasses systems using deep learning to support blind users in real-time scene understanding [11]. Multi-sensory guidance approaches combining YOLO-based object detection with SLAM have also shown promise for indoor navigation assistance [12,13]. None of these solutions provides a unified integrated platform combining face recognition, scene understanding, emergency alerts, and hands-free bilingual voice interaction at an affordable price point [14].

### 2.2. Face Recognition Techniques

Recent studies emphasize that facial recognition in unconstrained environments—characterized by varying poses, occlusions, and unpredictable lighting—requires highly robust architectures. InsightFace, driven by the Additive Angular Margin Loss (ArcFace) [15], has emerged as a dominant state-of-the-art solution for such challenges. Comparative evaluations by Fu et al. [16] demonstrated that ArcFace maintains superior discriminative power and stability under fluctuating image quality. In contrast, traditional Euclidean distance-based models like FaceNet [17] experience a notable drop in accuracy when faced with extreme intra-class variations and degraded image conditions. While FaceNet and its variants can achieve high accuracy in constrained, domain-specific scenarios [18,19], margin-based models like InsightFace consistently offer better generalization and robustness for the imperfect, single-shot images typical of real-world assistive applications.

Beyond verification accuracy, real-time processing speed is a critical requirement for mobile deployment. For visually impaired users, delayed auditory feedback degrades usability, making low inference latency as essential as recognition precision. Addressing these edge-deployment constraints, Luo et al. [20] proposed an optimized system combining efficient object detection with InsightFace, proving its capability to deliver high accuracy with minimal latency in real-time scenarios. Similarly, comprehensive benchmarks by Serengil and Ozpinar [21] confirm that margin-based pipelines can be highly optimized for edge devices without severe accuracy trade-offs. Given that visually impaired users rely on single, unconstrained image captures where both precision and immediate feedback are paramount, these findings highlight InsightFace as the most suitable candidate. However, the varying performance of these architectures across different environments directly motivates our empirical evaluation on the VGGFace2 subset, ensuring the selected model provides the optimal balance of accuracy and speed specifically for the Munir application.

### 2.3. Smart-Glasses Platforms

Open-source platforms such as the Omi Glass Dev Kit have recently emerged, providing developer-friendly hardware with programmable firmware that enables custom application development without proprietary lock-in [22]. This represents a 35× cost reduction versus commercial alternatives while maintaining essential functionality for assistive applications. Smart glasses equipped with inertial sensors have also demonstrated utility for activity classification using deep residual networks [23].

### 2.4. Comparative Analysis and Gap Identification

Existing assistive technologies exhibit three critical gaps: (1) Fragmentation—technologies address isolated challenges rather than providing integrated support; (2) Limited Multilingual Support —most solutions offer partial support for non-English languages; (3) Cost Barriers—commercial devices ($3500–$4250) are prohibitively expensive. Table 1 presents a detailed cost breakdown of the Munir system, distinguishing between one-time hardware costs, recurring API fees, and estimated maintenance expenses.

The estimated hardware acquisition cost of $610 covers the Omi Glass Dev Kit only. The Munir mobile application is freely downloadable, and no dedicated smartphone purchase is required as the system targets devices already owned by users. Google Cloud Vision API usage for OCR incurs no additional cost under standard free-tier allocations. Emergency SMS alerts are delivered via the user’s existing mobile carrier and are subject to standard SMS rates, which vary by provider and region. Object detection and scene description via the OpenAI GPT-5.2 API involve usage-based charges estimated at approximately $10 per month at typical assistive usage patterns. Annual hardware maintenance is estimated at 10–15% of the hardware acquisition cost, consistent with standard electronics maintenance benchmarks, yielding an estimated $61–$92 per year. Routine software maintenance costs are negligible given the open-source nature of the underlying frameworks (Flutter, InsightFace) and the absence of proprietary licensing fees. Accordingly, the total first-year cost is estimated at approximately $806, comprising the $610 hardware cost, $120 in API fees, and $76 in maintenance; annual recurring costs thereafter are estimated at approximately $196. Table 2 presents a systematic benchmarking of Munir against leading commercial and software-based solutions, highlighting how Munir addresses these gaps by combining a comprehensive feature set with economic accessibility.

## 3. Proposed System Architecture

**Figure 1:**
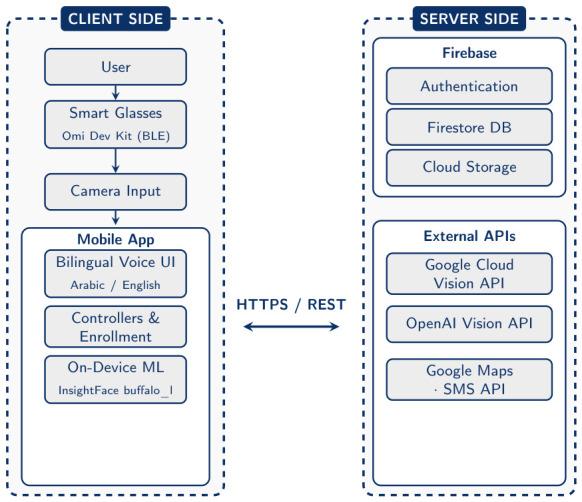
Munir client–server architecture with unified multimodal processing, on-device biometric isolation, and cloud service integration.

Munir employs a client–server architecture partitioning functionality between a mobile client and cloud backend. Figure 1 illustrates the system’s high-level architecture.

The mobile application coordinates: (1) smart glasses via Bluetooth Low Energy; (2) on-device InsightFace inference for face recognition; (3) an assistive voice-driven interface; (4) cloud services for authentication, data persistence, and API-based functionality.

**Figure 2:**
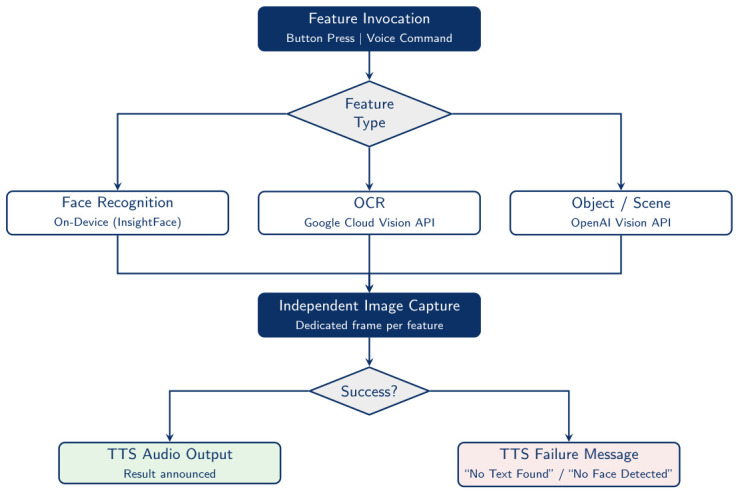
Feature invocation, dynamic routing, and outcome handling flow in Munir.

As illustrated in Figure 2, each assistive feature is invoked through one of two independent pathways: a dedicated button within the mobile application, or a voice command issued via the smart glasses. Vision-based features—face recognition, OCR, object recognition, and scene description—each trigger an independent image capture upon invocation, ensuring no shared input frame across features. Processing outcomes are communicated via Text-to-Speech: unrecognized faces are announced as “Unknown Person”, absent faces trigger a “No Face Detected” response, unreadable text produces a “No Text Found” message, and equivalent audio feedback is applied across all remaining features.

### 3.1. Hardware Platform

#### 3.1.1. Smart Glasses Hardware

**Figure 3:**
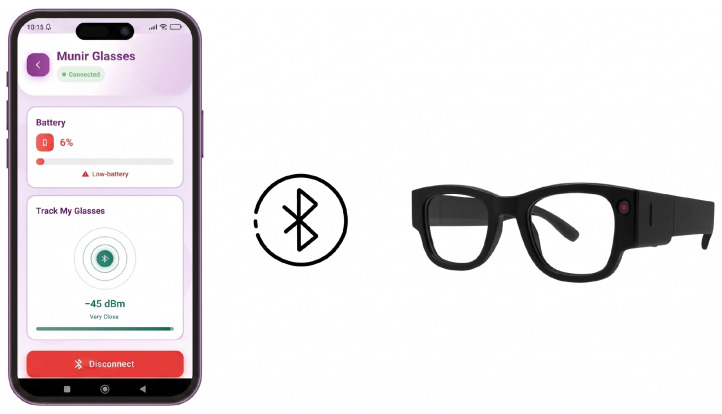
Omi Glass Dev Kit used as the smart glasses hardware platform in Munir, featuring an integrated camera, microphone, and Bluetooth 5.0 connectivity.

The Omi Glass Dev Kit serves as the hands-free hardware platform (Figure 3), built around the Seeed Studio XIAO ESP32-S3 Sense microcontroller (Seeed Technology Co., Ltd., Shenzhen, China) featuring a dual-core processor (up to 240 MHz) with OPI PSRAM. The device integrates an onboard camera module and supports 2.4 GHz Wi-Fi and Bluetooth Low Energy (BLE) connectivity. It is powered by a distributed LiPo battery configuration—six 150 mAh cells and one 250 mAh cell—totaling at approximately 1150 mAh, and is housed in a custom 3D-printed frame with standard glasses hinges. Released under the CC BY-SA 4.0 open-source license, the platform enables extensive customization and eliminates vendor lock-in. Battery life was evaluated over ten continuous usage sessions under active BLE connection, camera capture, and audio streaming, each starting from a full charge (100%). The device sustained an average operational duration of 4.5 h before requiring recharge.

#### 3.1.2. Mobile Device Requirements

Munir targets mid-range smartphones (Android 8.0+ or iOS 13.0+) with quad-core 2.0+ GHz processor, 3 GB+ RAM, 8 MP+ camera, and Bluetooth 5.0. These specifications are met by most smartphones manufactured from 2020 onwards, ensuring economic accessibility globally. All system evaluations reported in this paper were conducted on a Xiaomi Redmi 13 (MediaTek Helio G91-Ultra, Octa-core 2.2 GHz, 8 GB RAM, Android 14/Xiaomi HyperOS), which satisfies the minimum target specifications above.

### 3.2. Software Architecture

The mobile application is built using the Flutter framework with a single Dart codebase targeting both Android and iOS. The application follows a Model-View-Controller (MVC) pattern. Firebase Backend-as-a-Service provides authentication, Cloud Firestore database, and encrypted storage with security rules enforcing user-specific access control.

### 3.3. Bluetooth Low Energy Communication Architecture

The BLE stack operates over a dedicated GATT service with six characteristics: (1) a Photo Data characteristic for streaming JPEG frames from glasses to phone in 200-byte notify chunks; (2) a Photo Control characteristic for receiving capture commands from phone to glasses; (3) an Audio Data characteristic for continuous PCM microphone streaming, paused during photo transfer; (4) an Audio Codec characteristic exposing the active codec; (5) a User ID characteristic for the authenticated session string; (6) a Command Result characteristic notifying the phone of command outcomes. Battery level is reported through a separate GATT Battery Service. An MTU of 517 bytes is negotiated at connection setup, with a connection interval of 20–40 ms.

### 3.4. On-Device Machine Learning Strategy

A core design principle is that face recognition executes entirely on-device, ensuring: (1) real-time responsiveness; (2) offline functionality; (3) biometric privacy with no cloud transmission of facial data; (4) zero marginal cost per inference. The InsightFace buffalo_l model generates 512-dimensional L2-normalized embeddings, and identity matching is performed via cosine similarity against the user’s registered contact gallery.

## 4. Experimental Methodology

This section details the empirical framework established to evaluate and select the most suitable face recognition architecture. The methodology is designed to prioritize both recognition accuracy and computational efficiency, ensuring the system remains responsive under the hardware constraints of mobile edge devices.

### 4.1. Dataset Selection and Preparation

To ensure a robust evaluation, a curated subset of the VGGFace2 dataset [24] was utilized. The 540 identities were selected as the first 540 folders in alphabetical order from the dataset. For each identity, the first image (alphabetically) was assigned to the gallery, and all remaining 11 images served as probe queries, yielding 540 gallery images and 5940 probe images. Unlike laboratory-captured datasets, VGGFace2 is characterized by large variations in pose, age, lighting, and occlusion, effectively mimicking the unconstrained, real-world conditions that a mobile assistant would encounter. This sample size aligns with established benchmarking protocols [25], providing a statistically significant population to assess model generalization. All images underwent a standardized pre-processing pipeline where, beyond simple resizing to 224×224 pixels, geometric face alignment was implemented using an OpenCV-based face detector via the DeepFace framework, applied consistently across all three evaluated models. Model weights were loaded from official pre-trained repositories through DeepFace, and all inference speed measurements were conducted on Google Colab with a T4 GPU. Using predicted facial landmarks, each face was rotated and centered to a canonical pose to mitigate the impact of head pose variations, which are inherent in natural, non-professional captures.

### 4.2. Evaluation Protocol

**Figure 4:**
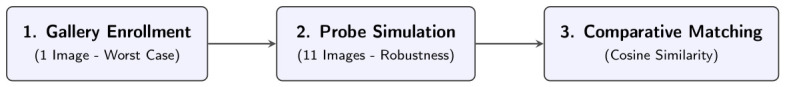
The systematic evaluation pipeline highlighting the single-image stress test and matching logic.

To ensure the selection of an architecture that balances high discriminative power with real-time efficiency, a systematic evaluation protocol was established. This framework is designed to test each model’s ability to generalize across “in-the-wild” facial variations while adhering to the hardware constraints of mobile deployment. Specifically, three prominent architectures were benchmarked: InsightFace buffalo_l, FaceNet512, and FaceNet (Original). By employing a closed-set identification protocol, the relative discriminative power of each architecture is compared under identical conditions. The protocol follows a structured pipeline (Figure 4) that transitions from database establishment to comparative analysis. A critical aspect of this protocol is the Single-Gallery-Image constraint; by utilizing only one reference image per person in the gallery, a worst-case scenario is intentionally simulated. This establishes a performance lower-bound, ensuring that if a model achieves high accuracy with minimal reference data, its robustness is improved even when real-world captures are sub-optimal. The methodology is summarized in the following three-step process:

1. Gallery Enrollment: Establishes a reference point for each identity by extracting a single high-dimensional embedding to act as a permanent digital signature. This single-shot setup tests the model’s ability to extract robust features from minimal data.
2. Probe Simulation: Subjects the models to a diverse set of query images (11 per identity) characterized by challenging lighting, varied poses, and occlusions to test recognition consistency across natural environments.
3. Comparative Matching: Utilizes cosine similarity to measure feature proximity and record performance metrics. Cosine similarity was specifically chosen for its illumination invariance and its ability to support instant, retraining-free registration, which is essential for the dynamic scaling of the Munir contact database.

No similarity threshold was applied in this model selection experiment, as the objective is to compare the relative discriminative power of the three architectures rather than to evaluate open-set rejection capability. Applying a fixed threshold would introduce model-specific bias, as optimal thresholds differ across architectures and embedding spaces. Identity prediction was therefore performed via argmax over cosine similarity scores. Regarding pre-training data, InsightFace buffalo_l was pre-trained on WebFace600K [26], which has no overlap with the VGGFace2 evaluation subset. FaceNet and FaceNet512 were pre-trained on VGGFace2; notably, InsightFace buffalo_l achieved the highest accuracy (91.52%) despite this asymmetry, further supporting its selection. Open-set evaluation with a rejection threshold is conducted in Section 6 using the SCface dataset. All benchmarking experiments were conducted using Python 3.12.0, DeepFace 0.0.93, and InsightFace 0.7.3 on Google Colab with a T4 GPU. Model weights for all three architectures were loaded from their official pre-trained repositories through the DeepFace framework. The mobile application was developed using Flutter 3.32.7 (Dart 3.8.1).

### 4.3. Performance Metrics

To provide a multidimensional analysis of the models’ recognition reliability and ensure their suitability for the Munir application, four complementary metrics were employed. These metrics evaluate the model’s discriminative power and statistical stability:

- Top-1 Identification Accuracy: This represents the primary success rate, calculated as the percentage of query images for which the highest-similarity gallery match corresponds to the correct ground-truth identity. In an assistive context, this reflects the system’s ability to provide the correct name to the user on the first attempt.
- Precision and Recall: These metrics assess the model’s behavior in a multi-class environment. Precision measures the fidelity of the system’s predictions, indicating the probability that a specific identity assignment is correct. Recall assesses the model’s ability to retrieve the correct identity from the gallery across various challenging query conditions. Both metrics are computed using macro-averaging across all 540 identity classes under the closed-set protocol, assessing per-class prediction consistency without bias toward frequent classes.
- Macro-averaged F1-Score: Given the multi-class nature of the 540 identity subset, the F1-Score serves as the harmonic mean of Precision and Recall. The macro-averaging approach treats all identities equally, ensuring that the model performs consistently across all registered contacts without bias toward specific classes.

### 4.4. Model Selection and Results Analysis

Upon the completion of the evaluation protocol, the benchmarked architectures demonstrated a clear performance hierarchy. The results, as summarized in Table 3, provide the empirical evidence required to finalize the core engine for the Munir system. The data reveals that while legacy architectures like FaceNet offer a basic level of recognition, they fail to maintain the necessary reliability under the Single-Gallery-Image (SGI) constraint.

The selection of InsightFace buffalo_l is justified by its demonstrated ability to overcome feature sparsity. Achieving a Top-1 Identification Accuracy of 91.52%—outperforming FaceNet512 by 25.9 percentage points—its ArcFace backbone effectively extracts invariant digital signatures from a single reference image. This level of accuracy ensures that the Munir application can provide dependable identification even in unconstrained environments with minimal enrollment data.

Furthermore, the F1-Score (89.71%) and the balance between Precision (89.20%) and Recall (91.44%) confirm that the model minimizes both misidentifications and retrieval failures. This balance is critical for a visually impaired user, for whom the reliability of identity announcements is the highest priority to ensure safe and confident social interactions. Consequently, InsightFace buffalo_l is chosen as the primary recognition engine, providing the necessary robustness to ensure consistent performance across all 540 registered identities.

## 5. System Implementation and Features

Munir integrates ten core capabilities into a unified accessible interface, providing TTS audio feedback for all user interactions.

### 5.1. Application Interface

**Figure 5:**
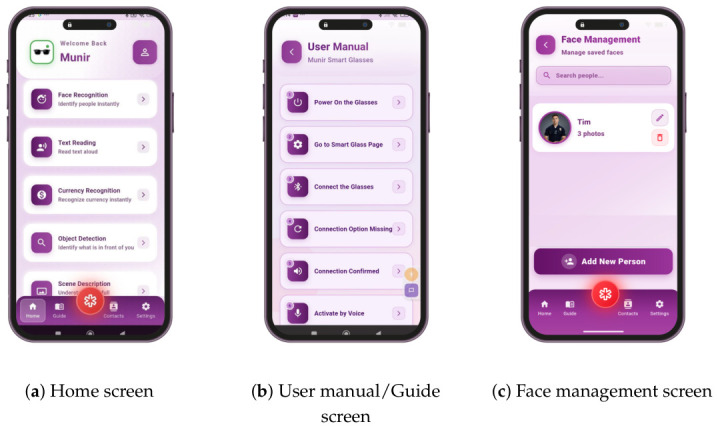
Munir mobile application screens. (**a**) Main dashboard showing core assistive features. (**b**) Step-by-step user manual depicting the initial six of seventeen smart glasses pairing and configuration steps. (**c**) Face management interface for registering and managing saved faces.

Figure 5 presents the main application screens illustrating the home dashboard, user guide, and face management interface. The interface applies a consistent purple design language throughout to signal assistive intent and brand identity. All interactive elements meet the WCAG 2.1 Level AA minimum touch-target size of 44 × 44 dp [27], and screen-reader labels are attached to every widget. A floating action button (bottom right of each screen) provides single-tap access to the voice-command overlay without requiring further navigation.

The Home screen (Figure 5a) serves as the main dashboard, offering single-tap access to all eight system features across the main menu and navigation bar. The User Manual screen (Figure 5b) presents a seventeen-step guide covering the complete pairing and configuration process, from initial power-on to glass power-off. Upon accessing the screen, the system automatically vocalizes all steps sequentially via Text-to-Speech (TTS). The Face Management screen (Figure 5c) allows users to register, update, and delete saved faces, enabling the system to recognize individuals upon detection, as described in Section 5.3.

### 5.2. Adaptive Pre-Processing Layer

The Munir face recognition architecture is built on the InsightFace buffalo_l model, utilizing a ResNet-50 backbone with ArcFace for extracting 512-dimensional L2-normalized embeddings. To ensure high reliability in the unconstrained environments typical of assistive technology, the pipeline incorporates an Adaptive Pre-processing Layer specifically designed to handle orientation inconsistencies and device-specific capture angles.

**Figure 6:**
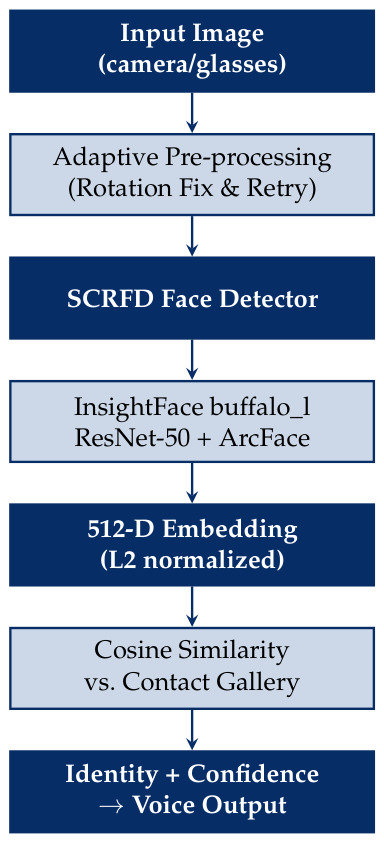
Munir face recognition pipeline with adaptive rotation pre-processing.

As illustrated in Figure 6, the system implements a Multi-Angle Retry Protocol to mitigate detection failures caused by motion or incorrect device orientation. Upon receiving an image, the system first performs EXIF orientation correction using the bakeOrientation method. If the initial SCRFD detection pass fails to localize a face, the system programmatically rotates the image in $90^{\circ }$ and $270^{\circ }$ increments, re-running the detection engine for each rotation. This ensures successful recognition even when the user captures images in landscape mode or at non-canonical angles.

### 5.3. Face Management Interface

**Figure 7:**
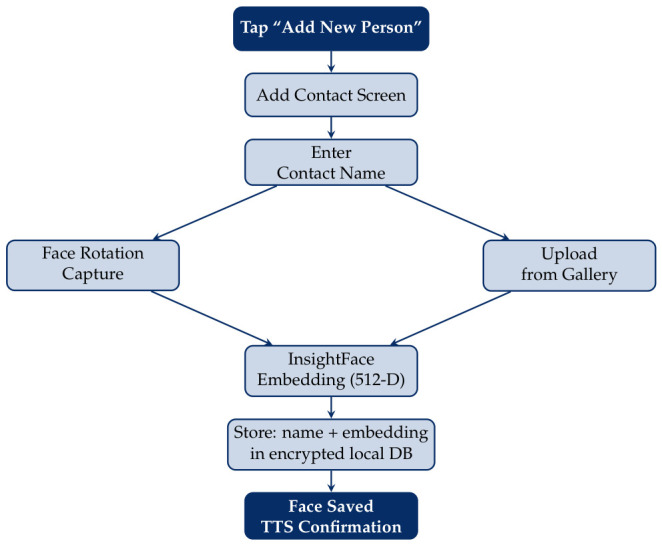
Munir Face Management workflow: tap-initiated face addition with rotation capture or direct image upload, InsightFace embedding generation, and encrypted on-device storage.

Beyond real-time identification, Munir provides a dedicated Face Management module that allows users to build and maintain their collection of saved faces through the application interface. This module enables users to register new entries, update existing information, and delete records to ensure the accuracy of the recognition system. Figure 7 illustrates the registration and management workflow.

The Face Management workflow operates as follows. The user initiates face addition by tapping the “Add New Person” button, which navigates to the Add Person screen. The user then types the person’s name and adds a facial image either via face rotation capture or direct image upload. The captured image is processed by InsightFace buffalo_l to extract a 512-dimensional L2-normalized embedding, which is stored alongside the person’s name in an encrypted local database, ensuring biometric data never leaves the device.

### 5.4. Optical Character Recognition

The OCR module processes both Arabic and English scene text by submitting captured frames to Google Cloud Vision API [28] via a Firebase Cloud Function. The API returns the recognized text, which is subsequently delivered to the user via bilingual Text-to-Speech output in the detected language, supporting both left-to-right English and right-to-left Arabic scripts.

### 5.5. Object Detection

Upon a voice command, a still frame captured from the smart glasses camera is submitted to the OpenAI GPT-5.2 API [29] with a prompt targeting single-object identification. The model returns a concise description of the foreground object, including its type, color, and key attributes, delivered to the user via Text-to-Speech.

### 5.6. Scene Description

Scene description provides environmental awareness for orientation tasks such as entering an unfamiliar space or navigating a public area. A captured frame is submitted to the OpenAI GPT-5.2 API [29] with a scene-oriented prompt, instructing the model to describe visible elements, their spatial relationships, and any notable obstacles, with the resulting narration delivered via Text-to-Speech.

### 5.7. Emergency Alert System

Upon activation—triggered by a button press on the smartphone or a voice command via the smart glasses—the system captures the user’s GPS coordinates and sends an SMS containing a Google Maps link to pre-configured emergency contacts. If no emergency contacts have been configured, the system prompts the user to add at least one before the feature can be used.

### 5.8. Task Reminders

The system supports four reminder patterns (one-time, daily, weekly, and monthly) delivered through multimodal notifications (audio and vibration). Reminder management is performed exclusively within the Munir application, where users can add reminders via voice commands (e.g., “Set a reminder for 8 AM daily to take medication”) or manually through the interface. Existing reminders can be modified or removed at any time, and a filtering feature allows users to efficiently browse reminders by schedule. To ensure user awareness, notifications are delivered simultaneously on both the smartphone and the smart glasses.

### 5.9. Bilingual Voice Interface

The system supports complete voice interaction in both Arabic and English across both the Munir application and the smart glasses. Wake word activation (“Hey Munir”) and keyword-based command routing are available on the smart glasses. The interface is fully compatible with TalkBack and VoiceOver screen readers.

### 5.10. Smart Glasses Integration

The Munir application communicates with the smart glasses exclusively over Bluetooth Low Energy (BLE), where the application acts as the GATT client and the glasses as the server. Upon establishing a connection, the application transmits a unique user identifier to the glasses to initiate the session. Subsequently, the glasses begin continuously streaming audio from the built-in microphone to the application in 200-byte chunks for real-time voice command processing. To optimize data throughput, an MTU of 517 bytes is negotiated at connection setup, with a connection interval of 20–40 ms to balance latency and power consumption. Battery levels are reported via a standard GATT Battery Service, while the Received Signal Strength Indicator (RSSI) is monitored client-side every 3 s to provide a proximity indicator for the user. To issue a command, the user vocalizes the wake word “Hey Munir,” followed by a specific instruction—such as “Who is in front of me?” or “Read the text.” Alternatively, pressing the physical button on the glasses triggers a comprehensive scene description, narrating all detected elements in the surroundings. Upon command reception, the glasses temporarily suspend audio streaming to capture and transmit an image to the application in 200-byte segments until a 0xFFFF end-of-image signal is detected, after which audio streaming automatically resumes. Notably, audio and image data are never transmitted simultaneously to ensure reliable BLE throughput and prevent data congestion. If the BLE connection is interrupted, the glasses automatically resume advertising to facilitate seamless reconnection without manual intervention.

## 6. Performance Evaluation and Benchmarking

### 6.1. Face Recognition Evaluation

#### 6.1.1. Dataset and Evaluation Setup

The face recognition pipeline of Munir was evaluated using two complementary datasets: the SCface surveillance cameras face database for closed-set recognition evaluation, and the Labeled Faces in the Wild (LFW) dataset [30] for open-set impostor probe evaluation.

##### SCface Dataset

SCface [31] contains 4160 images of 130 subjects captured in uncontrolled indoor environments using five surveillance cameras of varying quality, making it a well-established benchmark for real-world face recognition research under low-resolution and unconstrained conditions. Three representative images per subject—a frontal view and bilateral profile views (left and right)—were selected for gallery enrollment through the Munir application, consistent with the multi-angle onboarding protocol described in Section 5.3. The remaining 13 images per subject were assigned as probe queries across four evaluation conditions: 7 images capturing pose and camera angle variations to simulate smartphone camera capture, 5 images at varying distances to simulate smart glasses capture under sub-optimal conditions, and 1 infrared image to simulate low-light environments, yielding a total of 1690 probe images across all 130 subjects. Four evaluation conditions were defined, each corresponding to a real-world usage scenario for Munir:

- High-quality frontal—High-resolution frontal mugshot images representing optimal capture conditions, analogous to a close-range photograph taken by a standard smartphone camera. This condition serves as the performance upper bound.
- Pose variation—Lateral images at left and right profile angles, simulating natural off-axis orientations encountered when a visually impaired user photographs a person using Munir’s camera interface.
- Low-resolution surveillance—Images captured by surveillance cameras that exhibit low resolution due to camera quality and distance from the subject, as well as uncontrolled poses and illumination [31]. This condition simulates the image quality produced by the Omi Glass Dev Kit camera under sub-optimal capture distances.
- Infrared low-light—Images captured under infrared illumination, simulating low-light or night-time environments where visible-light cameras cannot produce reliable image quality.

##### LFW Dataset

The Labeled Faces in the Wild (LFW) dataset [30] is a publicly available benchmark comprising 13,233 face images of 5749 individuals collected from web sources under unconstrained conditions. For the open-set evaluation, 30 images were randomly sampled from 30 unique identities not present in the SCface-enrolled gallery, serving as impostor probes to assess the system’s ability to reject unregistered individuals.

Evaluation was conducted by submitting all dataset images to the Munir application’s on-device face recognition pipeline, which processes each input through the complete system pipeline: SCRFD face detection, adaptive rotation correction, InsightFace buffalo_l embedding extraction, cosine similarity matching against the enrolled gallery with threshold τ=0.40, and audio output generation.

#### 6.1.2. Closed-Set Evaluation Results

This section presents the quantitative evaluation results of the Munir face recognition pipeline on the SCface dataset, covering detection counts, recognition performance metrics, and system latency across all 130 subjects and 1690 probe images.

(i) Detection Counts

Table 4 reports the raw detection counts across all probe images.

The system correctly identified 1634 out of 1690 probe images, with zero false identity assignments across all evaluation conditions. All 56 missed identifications arose exclusively under the low-resolution surveillance condition, where degraded image quality produced less discriminative embeddings—a conservative failure mode appropriate for assistive deployment, where a missed identification is preferable to an incorrect one.

(ii) Recognition Performance

Table 5 summarizes the recognition performance metrics.

Munir achieved a recognition rate of 96.69% with 100% Precision and an F1-Score of 98.31%, confirming that no enrolled identity was ever incorrectly assigned to another person across all 1690 probe queries. The FRR of 3.31% reflects the system’s conservative cosine similarity threshold (τ=0.40), which prioritizes zero false accepts over minimizing false rejects. The False Accept Rate (FAR) is reported in Section 6.1.4 following open-set evaluation with impostor probes.

(iii) System Latency and Confidence

Table 6 reports the average end-to-end inference time and confidence score across all probe images.

The reported end-to-end latency of 3337 ms encompasses three stages: BLE image transmission from the smart glasses to the smartphone (2800 ms), on-device InsightFace inference (44.3 ms), and TTS audio output generation (493 ms), rather than model inference time alone.

#### 6.1.3. Per-Condition Analysis

**Figure 8:**
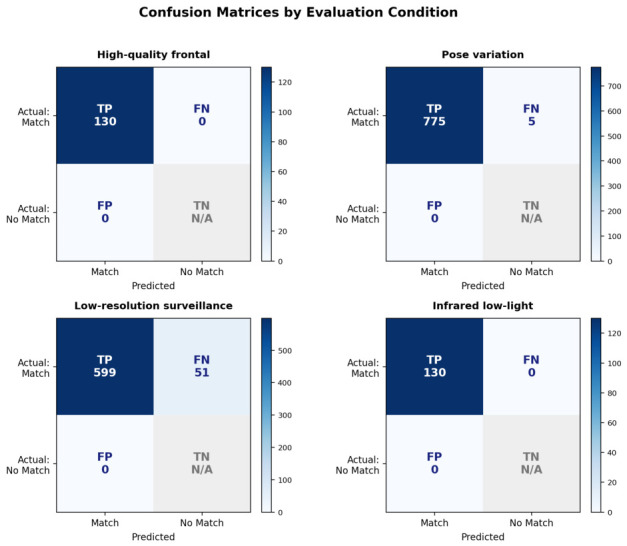
Confusion matrices of face recognition outcomes by evaluation condition. TP = True Positive (correct identification); FN = False Negative (missed identification); FP = 0 across all conditions; TN = N/A under the SCface evaluation protocol.

Figure 8 presents the confusion matrices of recognition outcomes for each evaluation condition. Each matrix reports True Positives (TPs) and False Negatives (FNs); False Positives (FPs) were zero across all conditions. True Negatives (TNs) are not applicable under the SCface evaluation protocol, as all probe images correspond to enrolled identities. Open-set evaluation including impostor probes is presented in Section 6.1.4.

Under high-quality frontal imaging, the system achieved 100% recognition (TP = 130, FN = 0), as expected given that these probe images share acquisition characteristics with the enrollment gallery. Under pose variation, the high recognition rate (TP = 775, FN = 5) validates Munir’s multi-angle enrollment strategy: registering three complementary views per identity—frontal, left profile, and right profile—allows the system to generalize across natural head orientations encountered in daily use.

The low-resolution surveillance condition produced the lowest recognition rate (TP = 599, FN = 51), consistent with the known challenge of cross-resolution face recognition in which low-quality probe images yield less discriminative embeddings [31]. Critically, the vast majority of False Negatives arose under this condition, with the system declining to output a match rather than returning an incorrect identity—a conservative failure mode appropriate for an assistive application where a missed identification is preferable to a wrong one.

Under infrared low-light conditions, perfect recognition was achieved (TP = 130, FN = 0), suggesting robustness under near-infrared imaging that extends Munir’s utility to night-time and low-ambient-light scenarios.

#### 6.1.4. Open-Set Evaluation with Impostor Probes

To validate the reported 0% FAR, a supplementary open-set evaluation was conducted by submitting 30 impostor probes drawn from the LFW dataset [30] to the Munir system, with the complete 130-identity SCface gallery remaining enrolled. Identity matching employs a cosine similarity threshold of τ=0.40; probe embeddings below this threshold are rejected as “Unknown Person”.

**Figure 9:**
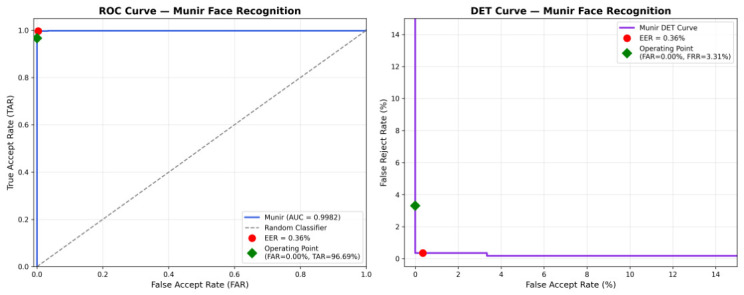
ROC curve (**left**) and DET curve (**right**) for Munir face recognition. The green diamond marks the system’s operating point and the red circle marks the Equal Error Rate (EER).

The system correctly rejected all 30 impostor probes, yielding a confirmed FAR of 0.00%. Impostor probe scores ranged from 0.080 to 0.244, all falling below the recognition threshold of τ=0.40. Genuine probes correctly identified by the system yielded scores ranging from 0.401 to 0.997, confirming complete score separation at the deployed threshold with no false accepts. Enrolled persons whose similarity score fell below τ=0.40 were conservatively rejected as “Unknown Person” (FRR = 3.31%), reflecting a deliberate design trade-off that prioritizes zero false accepts over minimizing false rejects—appropriate for an assistive application where an incorrect identity announcement poses greater risk than a missed identification. Figure 9 presents the corresponding ROC and DET curves, yielding an AUC of 0.9982 and an EER of 0.36%, confirming strong separability between genuine and impostor score distributions. At the deployed threshold τ=0.40, the system achieves FAR = 0.00% and FRR = 3.31%.

#### 6.1.5. Overall System Performance

Across the combined closed-set and open-set evaluation, Munir achieved a recognition rate of 96.69% with 100% Precision and an F1-Score of 98.31% on 1690 enrolled probe images, while correctly rejecting all 30 LFW impostor probes with a confirmed FAR of 0.00%. The FRR of 3.31% reflects the system’s conservative threshold strategy (τ=0.40), which prioritizes zero false accepts—a deliberate design choice appropriate for assistive deployment where an incorrect identity announcement poses greater risk than a missed identification.

#### 6.1.6. Comparison with Prior Work

Table 7 compares Munir against prior work evaluated on the same SCface dataset. Direct numerical comparison should be interpreted with caution given differences in evaluation protocols across studies. Specifically, the PCA baseline [31] and mpdCNN [32] used a single mugshot image for enrollment, whereas Munir employs three complementary views reflecting its intended multi-angle deployment conditions.

The SCface PCA baseline [31] achieved recognition rates below 10% at rank-1, reflecting the fundamental difficulty of low-resolution surveillance face recognition using traditional methods. Mishra et al. [32] improved upon this with a deep CNN architecture (mpdCNN), reaching 88.60%. Munir achieves 96.69%, an 8.09 percentage point improvement over the best result previously reported on this dataset under comparable evaluation conditions. This performance gain is primarily attributed to the integration of the InsightFace buffalo_l architecture with ArcFace loss, which optimizes the feature embedding space for high discriminative power even under the degraded image quality typical of surveillance captures. Furthermore, the achieved 100% Precision and 98.31% F1-Score confirm that the system produces no false identity assignments. This 0% False Accept Rate (FAR), confirmed through open-set evaluation with impostor probes (Section 6.1.4), represents a critical safety property for assistive deployment; it ensures the system prioritizes silent failure over providing incorrect auditory feedback, thereby eliminating potential social or safety-related errors for visually impaired users.

### 6.2. OCR Evaluation

#### 6.2.1. Dataset and Methodology

The OCR module was evaluated on the EvArEST (Everyday Arabic–English Scene Text) dataset [33], a publicly available benchmark comprising 510 real-world scene text images captured using mobile phone cameras under diverse indoor and outdoor conditions, including storefronts, street signage, product labels, and restaurant menus. Images with dimensions below 200×100 pixels were excluded as they contained single cropped words rather than a full scene text, as such images are insufficient for evaluating scene-level OCR performance. A random sample of 99 Arabic images and 100 English images was drawn from the remaining eligible images, consistent with established practices in OCR evaluation for assistive technology systems [34]. Evaluation was conducted by invoking the same Firebase Cloud Function endpoint used by the Munir application at runtime, which processes each image through Google Cloud Vision API and returns the recognized text. End-to-end inference time was recorded for each image to assess real-world responsiveness.

#### 6.2.2. OCR Performance Results

The OCR module was evaluated across 199 scene text images drawn from the EvArEST dataset, comprising 99 Arabic and 100 English images. Table 8 summarizes the evaluation results, where *n* denotes the number of images evaluated per language.

**Figure 10:**
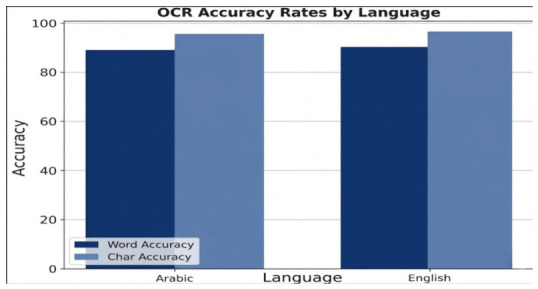
OCR Word Accuracy and Character Accuracy across Arabic and English scene text images drawn from the EvArEST dataset.

As shown in Table 8 and Figure 10, the OCR module achieved a Word Accuracy of 89.12% and Character Accuracy of 96.06% on an Arabic scene text, and 90.47% and 97.13% respectively on an English scene text. Both languages exhibit high and comparable accuracy, reflecting the bilingual optimization of the underlying Google Cloud Vision engine. Notably, the observed Word Accuracy for Arabic exceeds the 80% threshold commonly cited as the minimum acceptable performance for assistive OCR applications [34].

**Figure 11:**
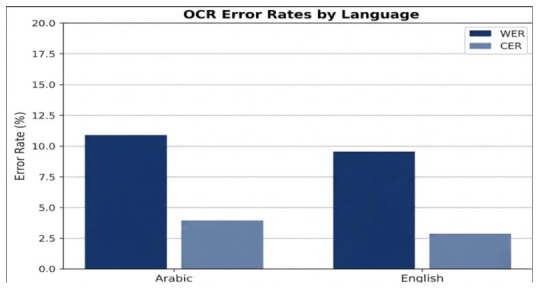
OCR Word Error Rate (WER) and Character Error Rate (CER) across Arabic and English scene text images.

As shown in Figure 11, the WER of 10.88% and CER of 3.94% for Arabic, as well as WER of 9.53% and CER of 2.87% for English, confirm that recognition errors are limited and character-level accuracy remains high across both languages. The lower CER relative to WER in both languages indicates that errors tend to affect isolated words rather than causing widespread character-level degradation.

As shown in Table 9, the average end-to-end inference time was 1818 ms for Arabic and 1774 ms for English inputs, reflecting the complete pipeline including image encoding, network transmission to the Firebase Cloud Function, Google Cloud Vision API processing, and response parsing. This latency is consistent with the responsiveness requirements of assistive OCR applications operating under real-world network conditions [34]. Overall, the Munir OCR module achieves a Word Accuracy above 89% and a Character Accuracy above 96% for both Arabic and English on real-world scene text images, demonstrating consistent bilingual performance across varied fonts, lighting conditions, and viewing angles typical of daily assistive use scenarios.

### 6.3. Task Reminder Evaluation

#### 6.3.1. Methodology

The task reminder module was evaluated through controlled functional testing, following established mobile application testing practices in which developers conduct manual verification of feature correctness through structured test cases [35,36]. Evaluation was conducted across 30 controlled reminder trials, distributed equally across three app states: foreground (application open and visible), background (application minimized), and closed (application fully terminated), with 10 trials per condition. For each trial, a reminder was scheduled at a predefined time and the delivery timestamp was recorded manually upon notification arrival. Two metrics were computed: notification delivery success rate, defined as the percentage of trials in which the notification was received, and timing deviation, defined as the absolute difference in seconds between the scheduled and actual delivery times.

#### 6.3.2. Results

Table 10 summarizes the evaluation results across the three app states.

As shown in Table 10, the reminder module achieved a 100% delivery success rate across all 30 trials and all three app states, confirming reliable notification delivery regardless of application lifecycle state. The overall mean timing deviation was 4.4 s (range: 0–12 s), which is within acceptable bounds for assistive reminder applications. Notably, foreground trials exhibited a higher average deviation (10.2 s) compared to background (1.8 s) and closed (1.2 s) conditions. This difference is attributable to the additional processing overhead introduced when the application is active: in the foreground state, Firebase Cloud Messaging delivers the notification as a data message routed through the Flutter message handler, which incurs additional processing latency before display. In contrast, background and closed states receive notifications directly from the Firebase Cloud Messaging infrastructure without passing through the Flutter application layer, resulting in near-immediate delivery. These results confirm the functional correctness and reliability of the Munir reminder module across real-world usage conditions.

## 7. Discussion

### 7.1. Performance and Methodology Analysis

The experimental results demonstrate a clear progression from theoretical model selection to practical system deployment. The initial benchmarking on the VGGFace2 subset was specifically designed as a worst-case stress test. By employing a Single-Gallery-Image protocol—the most restrictive condition in face recognition—the system was forced to identify subjects with minimal prior data. Despite these extreme constraints, the architecture achieved a robust baseline accuracy of 91.52%, proving that the InsightFace buffalo_l engine can maintain high discriminative power even under optimal data scarcity. The subsequent transition to the SCface dataset provided a validation of the fully integrated Munir system. The observed recognition rate of 96.69% on SCface is not a contradiction to the initial baseline, but rather a verification of the Multi-angle Onboarding Protocol. By registering three complementary facial views, the system effectively overcomes the pose-variance limitations identified during the worst-case tests. This dual-dataset validation underscores the reliability of the model: it is not only capable of performing under the data-poor conditions of the VGGFace2 stress test but also excels in the unconstrained, low-resolution environments typical of surveillance and smart-glass hardware. Furthermore, the 56 recorded missed identifications (False Negatives) are classified as low-severity operational issues, as a silent failure in assistive HCI is significantly safer than a false identification that could lead to social or physical risks for the visually impaired user.

### 7.2. Latency and Operational Turnaround

Regarding system responsiveness, it is essential to distinguish between inference latency and operational turnaround time. While the InsightFace engine achieves an optimized inference speed of 44.3 ms on the mobile processor, the total end-to-end system latency of 3337 ms accounts for the entire communication pipeline. This includes the Bluetooth Low Energy (BLE) transmission of image packets from the glasses to the smartphone, on-device inference, and final Text-to-Speech generation. This distinction confirms that while the core AI is highly efficient, the overall user experience is primarily governed by the hardware’s data-transfer protocols.

### 7.3. Privacy and Ethical Considerations

Munir adopts a tiered data handling architecture that separates biometric from non-biometric processing. Face recognition executes entirely on-device: biometric embeddings are stored in an encrypted local database and never transmitted to external servers, ensuring that facial data remains under the user’s exclusive control. Images captured in real time for recognition queries are processed in memory without persistent storage. For enrolled contacts, only the 512-dimensional L2-normalized embedding is securely stored locally; the original enrollment image is never retained. Cloud-dependent features (object detection and scene description via OpenAI GPT-5.2, and OCR via Google Cloud Vision API) transmit captured scene frames to external cloud services for processing. These images may incidentally contain bystander faces, though no facial recognition or biometric processing is performed on them by Munir; the face recognition feature operates exclusively against the user’s pre-enrolled contact gallery. Submitted frames may be temporarily retained in accordance with the provider’s data retention policies for abuse monitoring purposes before being automatically deleted [29]. Users are explicitly informed within the application of this distinction between on-device biometric processing and cloud-based features, enabling informed consent prior to use. Nonetheless, users are advised to exercise discretion when activating camera-based features in environments where bystander privacy is a concern. All data transmission between the mobile application and cloud services is encrypted via HTTPS/TLS. Local BLE communication between the smart glasses and smartphone operates over LE Secure Connections (BLE v4.2+), which provides hardware-accelerated AES-CCM 128-bit Link Layer encryption via the ESP32-S3 following ECDH P-256 key exchange, mitigating risks such as eavesdropping and Man-in-the-Middle attacks at the protocol level. Future iterations will explore fully on-device alternatives for scene understanding using lightweight on-device vision models based on architectures such as MobileNetV2 [37] to further reduce cloud dependency.

### 7.4. Economic Reach and Global Impact

The bilingual (Arabic/English) voice interface with WCAG 2.1 Level AA compliance addresses the needs of diverse global populations. Emergency alert functionality via SMS ensures reliability even in low-bandwidth conditions, unlike internet-dependent messaging. At an estimated first-year total cost of $806, Munir enables individual purchase or institutional distribution at a fraction of commercial alternative costs.

### 7.5. Limitations and Future Directions

Several limitations regarding the operational environment require acknowledgment. While the system demonstrated significant robustness against low-resolution surveillance captures, its performance remains susceptible to extreme environmental factors, such as total darkness exceeding the effective range of the infrared sensors. Furthermore, severe motion blur resulting from rapid head movements or high-speed subject displacement can still degrade recognition accuracy. Future research will focus on integrating automated image-quality assessment and more robust motion-compensation algorithms to maintain high reliability under these challenging dynamic conditions. Additionally, quantitative evaluation of the object detection, scene description, and voice interaction modules is planned for future work. A formal UAT study with visually impaired users and domain experts will also be conducted to assess system usability and real-world effectiveness.

## 8. Conclusions

This paper presented Munir, an integrated AI-powered assistive system combining a Flutter mobile application with BLE-connected smart glasses to provide comprehensive support for visually impaired individuals. Through a rigorous empirical evaluation of three pre-trained face recognition models on a curated VGGFace2 subset, InsightFace buffalo_l was identified as the highest-performing backbone, achieving a robust baseline accuracy of 91.52% under a “worst-case” Single-Gallery-Image protocol. The fully integrated system further demonstrated its efficacy on the SCface dataset, achieving a 96.69% recognition rate, validating the Multi-angle Onboarding Protocol’s ability to overcome pose and resolution challenges typical of unconstrained environments. Four key aspects distinguish Munir: (1) Comprehensive multimodal integration of ten essential capabilities—including face recognition, OCR, and scene understanding—within a unified bilingual voice interface; (2) systematic empirical model selection, with InsightFace buffalo_l identified as the highest-performing backbone through rigorous benchmarking against FaceNet512 and FaceNet; (3) privacy-preserving on-device ML ensuring biometric data remains locally encrypted and never transmitted to external servers; (4) cost-effective hardware, with an estimated first-year total cost of $806, representing a 4–5× cost advantage over commercial alternatives. Future work will focus on optimizing motion-compensation algorithms, expanding the suite of offline multimodal features, and conducting a formal UAT study with visually impaired users to ensure reliable, high-performance assistance in diverse and dynamic settings. 

## Figures and Tables

**Table 1:** Munir system cost breakdown.

Cost Component	Type	Frequency	Estimated Cost (USD)
Omi Glass Dev Kit	Hardware	One-time	$610
Mobile Application	Software	One-time	Free
OpenAI GPT-5.2 API	Cloud Service	Monthly	∼$10
Google Cloud Vision API	Cloud Service	Monthly	Free
Hardware Maintenance	Maintenance	Annual	$61–$92
First-Year Total			∼$806
Annual Recurring			∼$196

**Table 2:** Comparative analysis of Munir vs. state-of-the-art commercial assistive technologies.

Key Feature	Munir	OrCam MyEye	Envision	Seeing AI
Hands-Free Operation	✔	✔	✔	×
Face Recognition	✔	✔	✔	✔
Bilingual OCR	✔	✔	✔	✔
Color Detection	✔	✔	✔	✔
Object Recognition	✔	✔	✔	✔
Scene Description	✔	×	✔	✔
Emergency SOS	✔	×	×	×
Task Scheduling	✔	×	×	×
Smart-Glasses Interface	✔	✔	✔	×
Bilingual TTS (Ar/En)	✔	✔	✔	×
On-Device Processing	✔	✔	Partial	×
Total Features	10/10	7/10	8/10	5/10
Cost (USD)	$806 ^†^	$4250	$3500	Free *

* Seeing AI (tested January 2026) is a software-only solution requiring continuous smartphone handling. ✔ = fully supported; × = not supported. ^†^ Estimated first-year total cost; see Table 1 for full breakdown.

**Table 3:** Comparative performance analysis of facial recognition architectures under the SGI constraint.

Model	Accuracy	Precision	Recall	F1-Score
FaceNet (Original)	47.46%	62.18%	47.46%	51.69%
FaceNet512	65.61%	69.54%	65.61%	66.14%
InsightFace buffalo_l	91.52%	89.20%	91.44%	89.71%

**Table 4:** Detection counts from SCface closed-set evaluation.

Metric	Value
Total Probe Images	1690
True Positives (TPs)	1634
False Positives (FPs)	0
False Negatives (FNs)	56

**Table 5:** Recognition performance metrics on the SCface closed-set evaluation.

Metric	Value
Recognition Rate (RR)	96.69%
Precision	100.00%
Recall	96.69%
F1-Score	98.31%
False Reject Rate (FRR)	3.31%

**Table 6:** End-to-end latency breakdown and average confidence score on the SCface evaluation.

Pipeline Stage	Avg. Time (ms)
BLE Image Transmission (glasses → smartphone)	∼2800
On-Device InsightFace Inference	44.3
TTS Audio Output Generation	∼493
Total End-to-End Latency	3337
Avg. Confidence Score	0.68

**Table 7:** Benchmarking Munir against prior methods evaluated on the SCface dataset.

Study/System	Year	Approach/Backbone	Accuracy (RR)
Grgic et al. [31]	2011	PCA (Baseline)	<10.00%
Mishra et al. [32]	2021	mpdCNN (Deep Learning)	88.60%
Munir (Ours)	2026	InsightFace buffalo_l (ResNet-50) + ArcFace	96.69%

**Table 8:** OCR evaluation results on the EvArEST bilingual scene text dataset. *n* = number of evaluated images.

Language	*n*	Word Acc. (%)	WER (%)	Char Acc. (%)	CER (%)
Arabic	99	89.12	10.88	96.06	3.94
English	100	90.47	9.53	97.13	2.87

**Table 9:** Average end-to-end OCR inference time per image for Arabic and English inputs via the Munir Firebase Cloud Function.

Language	Avg. Inference Time (ms)
Arabic	1818
English	1774

**Table 10:** Task reminder functional evaluation results across three app states (10 trials per condition).

App State	Trials	Delivered	Success Rate (%)	Avg. Deviation (s)
Foreground	10	10	100.0	10.2
Background	10	10	100.0	1.8
Closed	10	10	100.0	1.2
Overall	30	30	100.0	4.4

## Data Availability

The curated VGGFace2 subset is derived from the VGGFace2 dataset [24], available for academic use. Evaluation code is available from the corresponding author upon reasonable request.

## Abbreviations

The following abbreviations are used in this manuscript:

AI	Artificial Intelligence
API	Application Programming Interface
AES	Advanced Encryption Standard
BLE	Bluetooth Low Energy
CA	Character Accuracy
CER	Character Error Rate
ECDH	Elliptic Curve Diffie–Hellman
ESP	Espressif Systems Platform
FAR	False Accept Rate
FN	False Negative
FP	False Positive
FRR	False Reject Rate
GPS	Global Positioning System
GATT	Generic Attribute Profile
HCI	Human–Computer Interaction
LFW	Labeled Faces in the Wild
ML	Machine Learning
MTU	Maximum Transmission Unit
MVC	Model-View-Controller
OCR	Optical Character Recognition
RR	Recognition Rate
RSSI	Received Signal Strength Indicator
SGI	Single-Gallery-Image
SMS	Short Message Service
SOS	Save Our Souls
TP	True Positive
TTS	Text-to-Speech
UI	User Interface
WA	Word Accuracy
WCAG	Web Content Accessibility Guidelines
WER	Word Error Rate
